# Supplementary material for: Modelling livestock infectious disease control policy under differing social perspectives on vaccination behaviour
Source: PLoS Comput Biol. 2022 Jul 14;18(7):e1010235. doi: 10.1371/journal.pcbi.1010235 (PMC9282555; doi:10.1371/journal.pcbi.1010235)
Supplement: S1 Text — (PDF) [file pcbi.1010235.s001.pdf]

Supporting Information: Modelling livestock infectious disease control policy under differing social perspectives on vaccination behaviour

Edward M. Hill[1,2*], Naomi S. Prosser[3], Eamonn Ferguson[4], Jasmeet Kaler[3], Martin J. Green[3], Matt J. Keeling[1,2], Michael J. Tildesley[1,2].

1 The Zeeman Institute for Systems Biology and Infectious Disease Epidemiology Research, School of Life Sciences and Mathematics Institute, University of Warwick, Coventry, United Kingdom.
2 Joint UNIversities Pandemic and Epidemiological Research, https://maths.org/juniper/.
3 School of Veterinary Medicine and Science, Sutton Bonington Campus, University of Nottingham, Leicestershire, United Kingdom.
4 School of Psychology, University Park, University of Nottingham, Nottingham, United Kingdom.

* Corresponding Author. Email: Edward.Hill@warwick.ac.uk

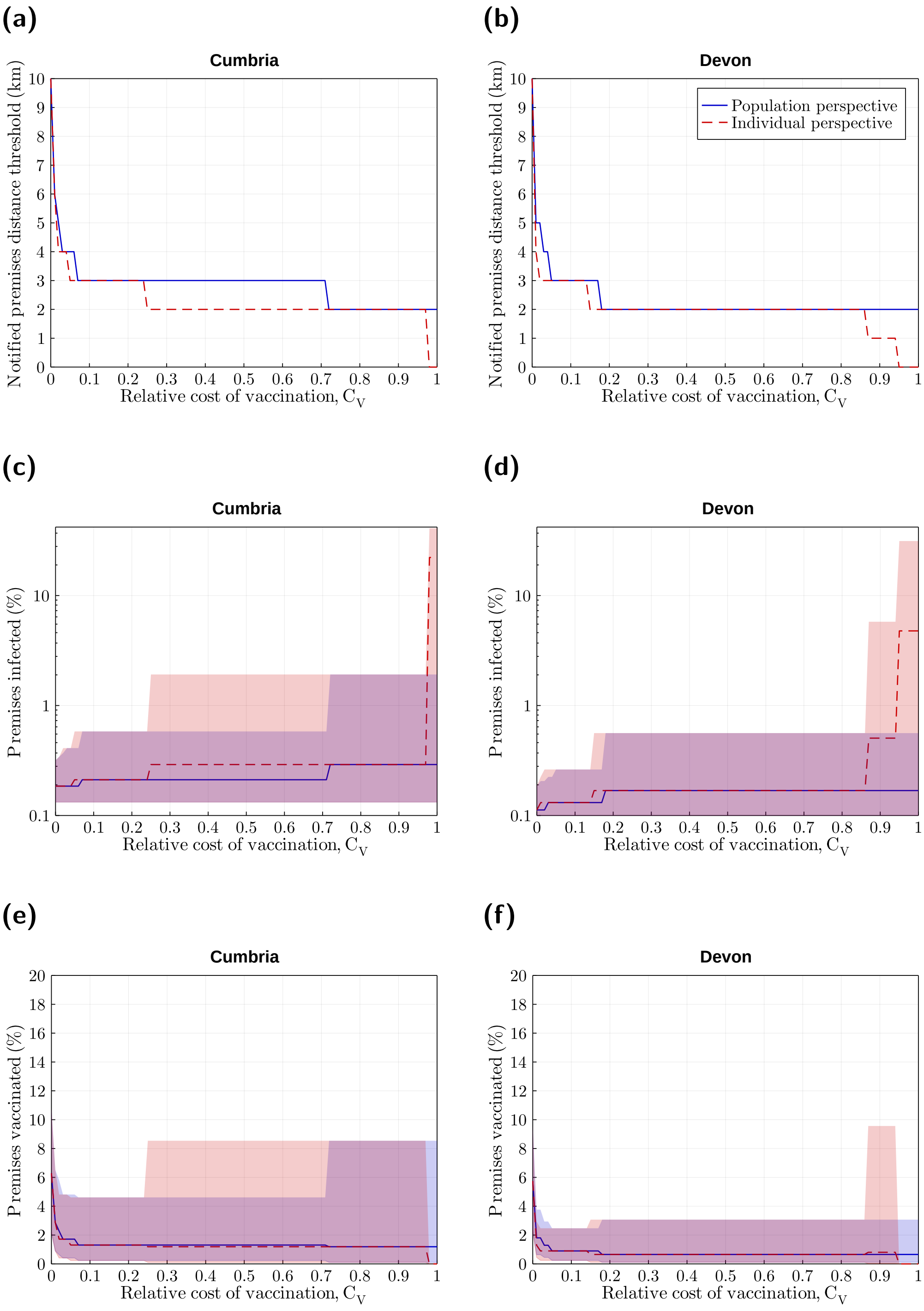

Fig A: For the 'alternate' pathogen, the reactive vaccination strategy that minimises the overall cost from either the population or individual perspectives, dependent upon the relative cost of vaccination versus infection. We performed 500 replicates per scenario. Vaccination at a premises was triggered by notification of infection within the specified distance threshold (no premises were initialised as either having already been vaccinated or in the 'never vaccinate' group). We identified the lowest median cost (with there being a distribution of costs from the ensemble of stochastic simulations) across the control scenarios from a population perspective (blue solid line) and an individual (farmer) perspective (red dashed line) for: **(a)** Cumbria; **(b)** Devon. We recall that the possible reactive vaccination distance thresholds were 0km to 10km inclusive (in 1km increments). Therefore, as it was not possible for intermediate distances between these integer values to be optimal, we obtained step-like line profiles. Under the identified optimum reactive vaccination strategy, we show medians (lines) and 95% prediction intervals (shaded regions) for: **(c,d)** the percentage of premises infected (note the y-axis is on a log scale); **(e,f)** the percentage of premises vaccinated.

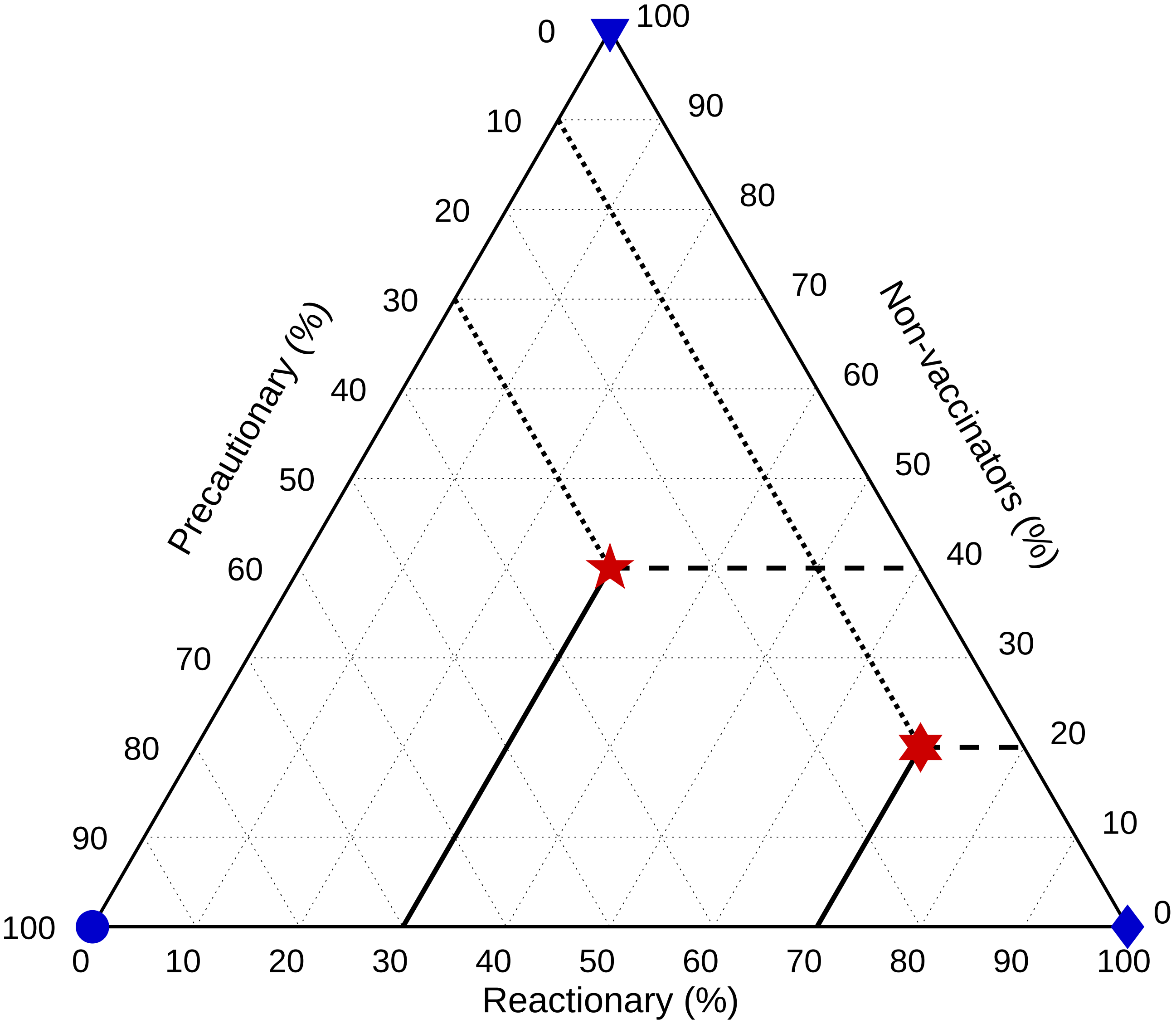

Fig B: Reading values from our ternary plots. In Figs 3-4 and Figs C-L, we use ternary plots to visualise epidemiological outputs produced under different population splits between our three vaccine behaviour groups (precautionary, reactionary, non-vaccinators). A ternary plot graphically depicts the ratios of three variables (that sum to a constant) as positions in an equilateral triangle. For a given marker on the plot, the percentage of the population in each vaccine group may be read as follows: Precautionary - from the marker move up and parallel to the right-hand axis, continuing until intercepting the left-hand axis and read off the intercept value (depicted by the dotted lines); Reactionary - from the marker move down and parallel to the left-hand axis, continuing until intercepting the bottom axis and read off the intercept value (depicted by the solid lines); Non-vaccinators - move from the marker horizontally to the right-hand axis, reading off the intercept value (depicted by the dashed lines). We illustrate this procedure with two examples: red pentagram representing a vaccine group composition of 30% precautionary, 30% reactionary and 40% non-vaccinators; red hexagram representing a vaccine group composition of 10% precautionary, 70% reactionary and 20% non-vaccinators. We also mark with blue markers the points corresponding to the vaccine group composition being comprised of a single group only: 100% precautionary (circle), 100% reactionary (diamond), 100% non-vaccinators (inverted triangle).

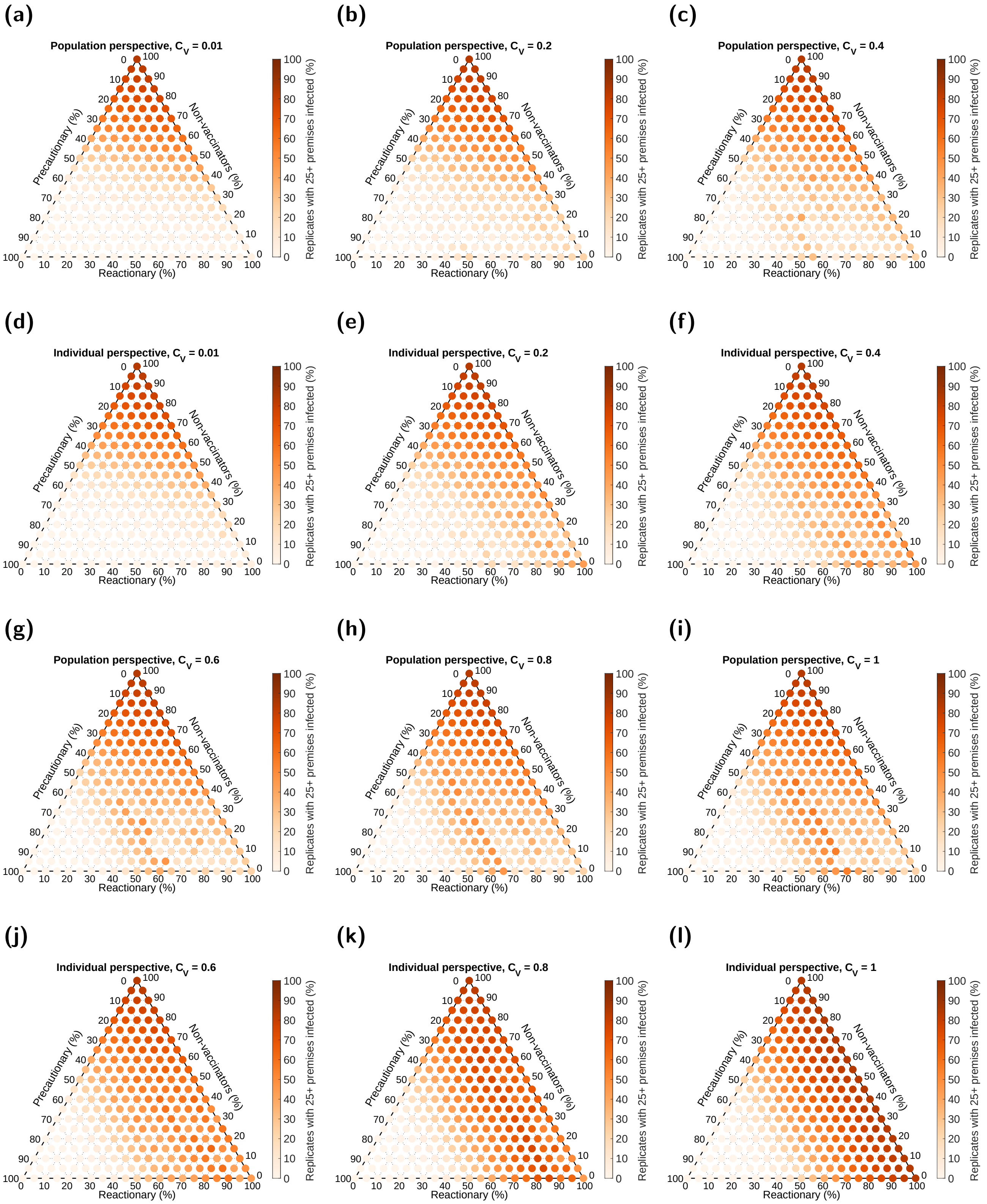

Fig C: Percentage of replicates resulting in 25 or more premises becoming infected under the reactive vaccination strategy minimising the cost in Cumbria. We performed 500 replicates per scenario. Light to dark shading corresponding to a larger percentage of runs having outbreaks with 25 or more premises infected. The ternary plots display infection outcomes for the reactive vaccination strategy identified as optimal (from population, individual perspectives) given a relative cost of vaccination, C_V, of: **(a,d)** 0.01; **(b,e)** 0.2; **(c,f)** 0.4; **(g,j)** 0.6; **(h,k)** 0.8; **(i,l)** 1. For an explanation on reading values on a ternary plot, see Fig B.

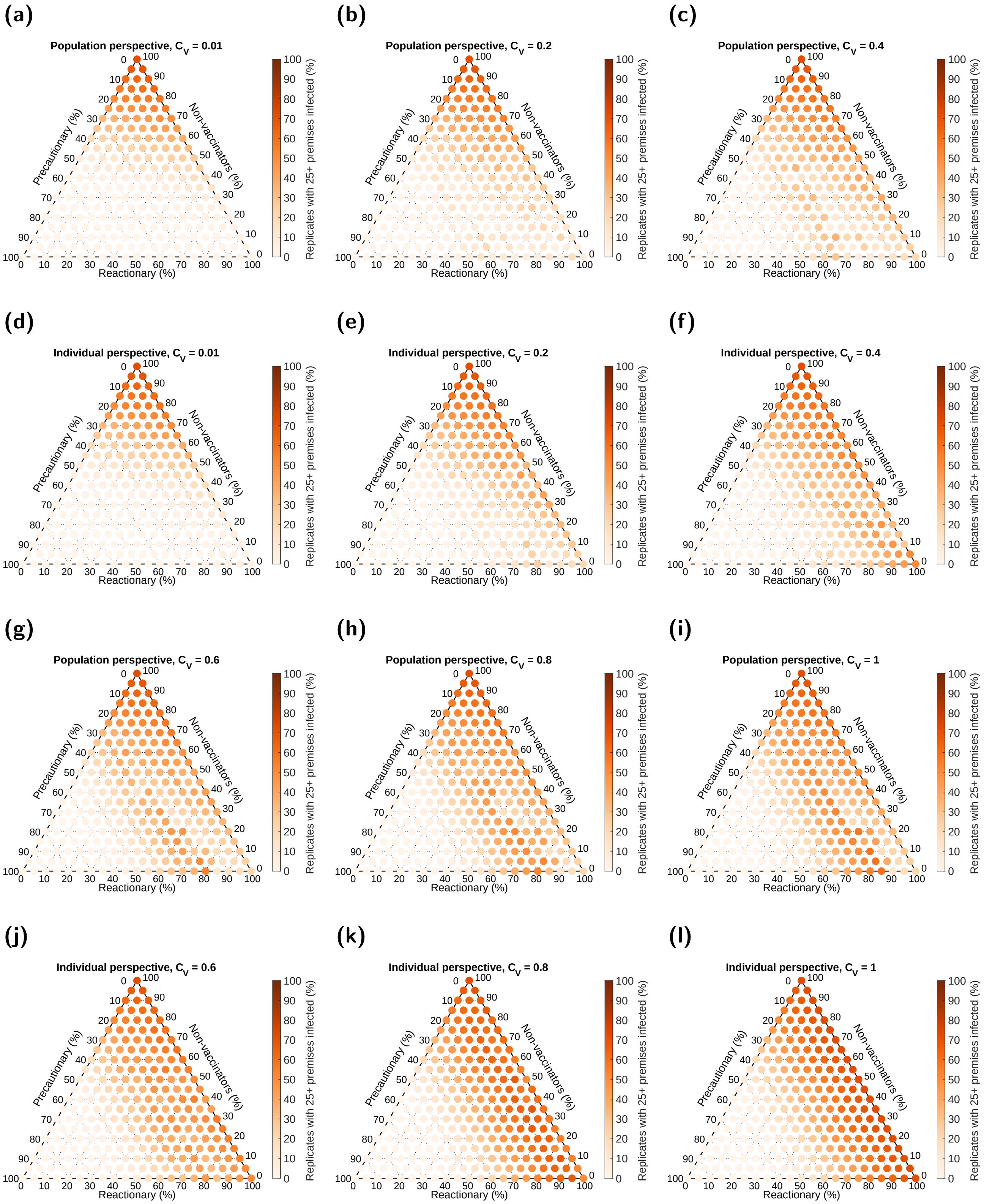

Fig D: Percentage of replicates resulting in 25 or more premises becoming infected under the reactive vaccination strategy minimising the cost in Devon. We performed 500 replicates per scenario. Light to dark shading corresponding to a larger percentage of runs having outbreaks with 25 or more premises infected. The ternary plots display infection outcomes for the reactive vaccination strategy identified as optimal (from population, individual perspectives) given a relative cost of vaccination, C_V, of: **(a,d)** 0.01; **(b,e)** 0.2; **(c,f)** 0.4; **(g,j)** 0.6; **(h,k)** 0.8; **(i,l)** 1. For an explanation on reading values on a ternary plot, see Fig B.

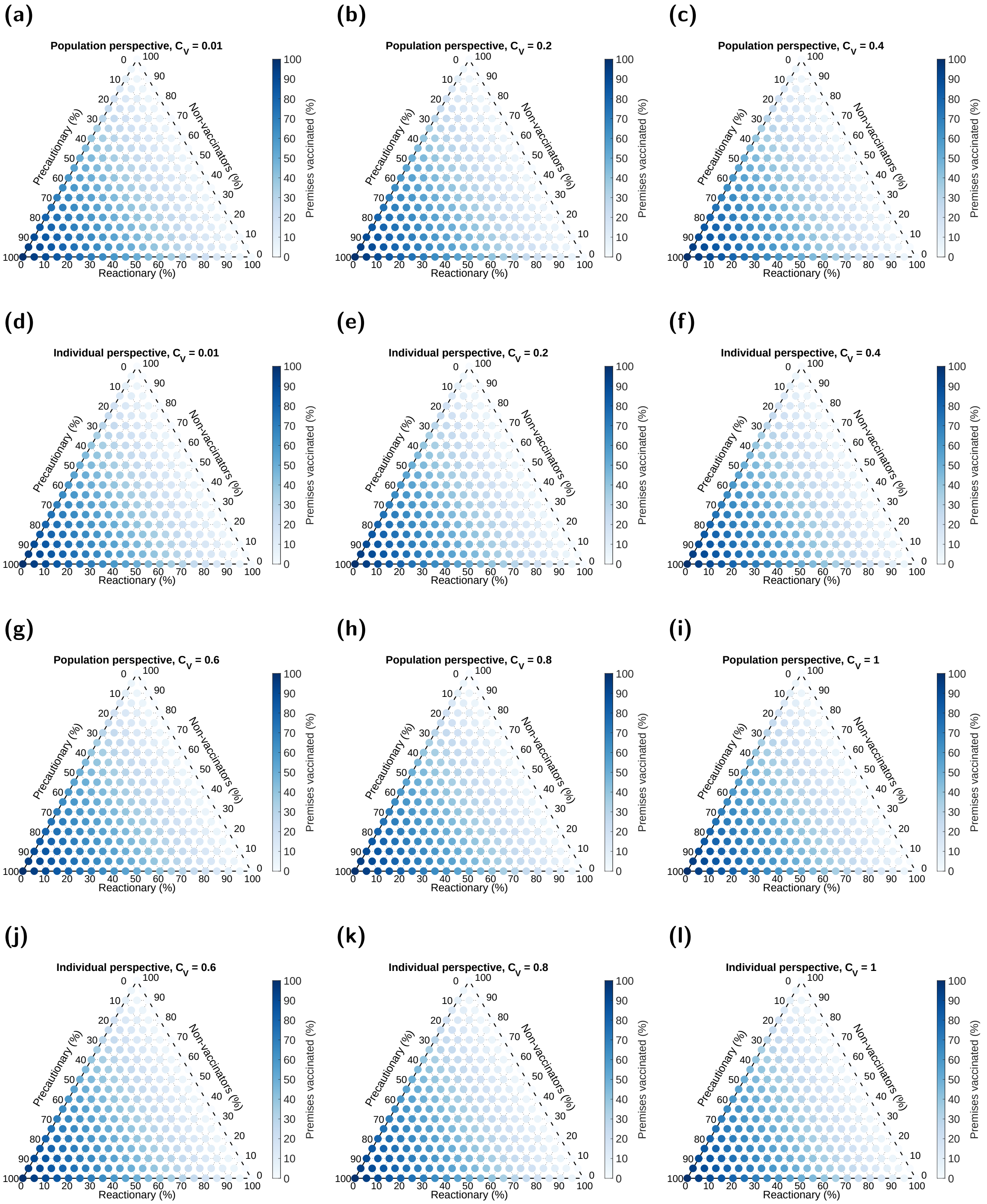

Fig E: Median percentage of premises vaccinated under the reactive vaccination strategy minimising the cost in Cumbria. We performed 500 replicates per scenario. Light to dark shading corresponding to a larger percentage of premises vaccinated. The ternary plots display vaccination outcomes for the reactive vaccination strategy identified as optimal (from population, individual perspectives) given a relative cost of vaccination, C_V, of: **(a,d)** 0.01; **(b,e)** 0.2; **(c,f)** 0.4; **(g,j)** 0.6; **(h,k)** 0.8; **(i,l)** 1. For an explanation on reading values on a ternary plot, see Fig B.

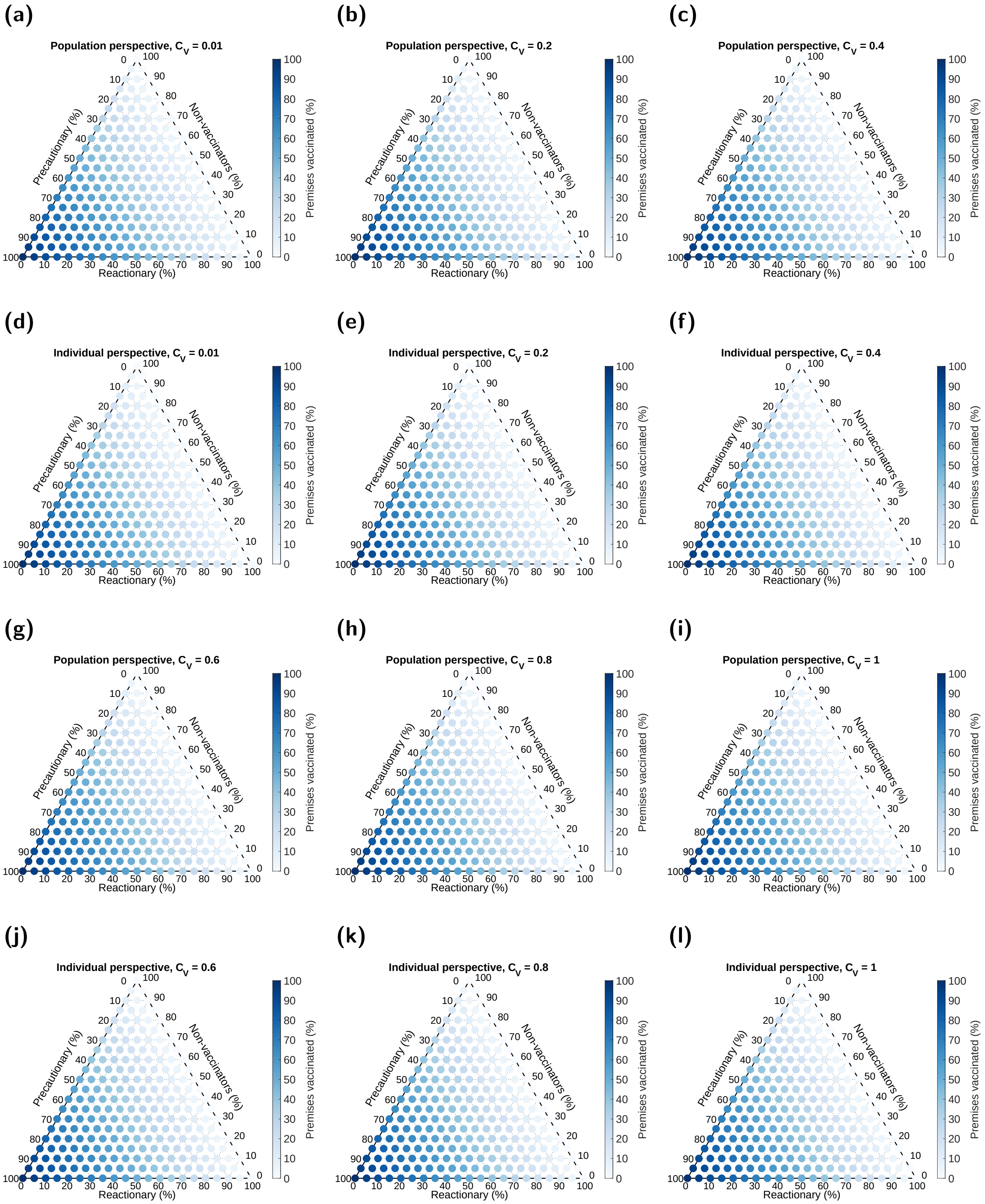

Fig F: Median percentage of premises vaccinated under the reactive vaccination strategy minimising the cost in Devon. We performed 500 replicates per scenario. Light to dark shading corresponding to a larger percentage of premises vaccinated. The ternary plots display vaccination outcomes for the reactive vaccination strategy identified as optimal (from population, individual perspectives) given a relative cost of vaccination, C_V, of: **(a,d)** 0.01; **(b,e)** 0.2; **(c,f)** 0.4; **(g,j)** 0.6; **(h,k)** 0.8; **(i,l)** 1. For an explanation on reading values on a ternary plot, see Fig B.

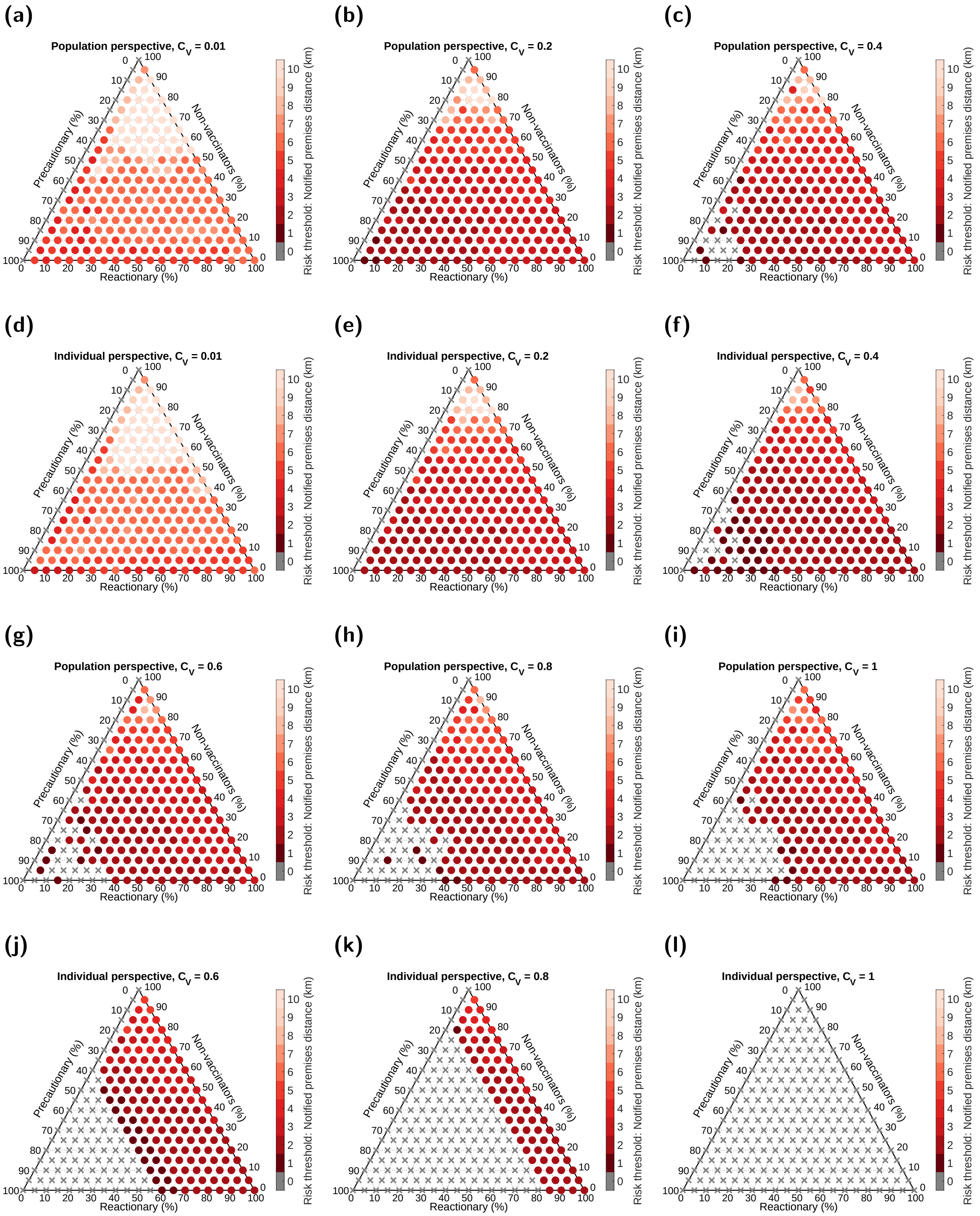

Fig G: Strategy that minimises overall cost in Cumbria for the 'alternate' pathogen, dependent on the perspective, relative cost of vaccination and vaccine group composition. We performed 500 replicates per scenario. Shading corresponds to the notification distance risk threshold d that returned the lowest median cost across the control scenarios (dark to light corresponding to increases in distance from 1km to 10km). Grey crosses correspond to no reactive vaccination occurring. The ternary plots display optimal strategies from the population and individual perspectives given a relative cost of vaccination, C_V, of: **(a,d)** 0.01; **(b,e)** 0.2; **(c,f)** 0.4; **(g,j)** 0.6; **(h,k)** 0.8; **(i,l)** 1. For an explanation on reading values on a ternary plot, see Fig B.

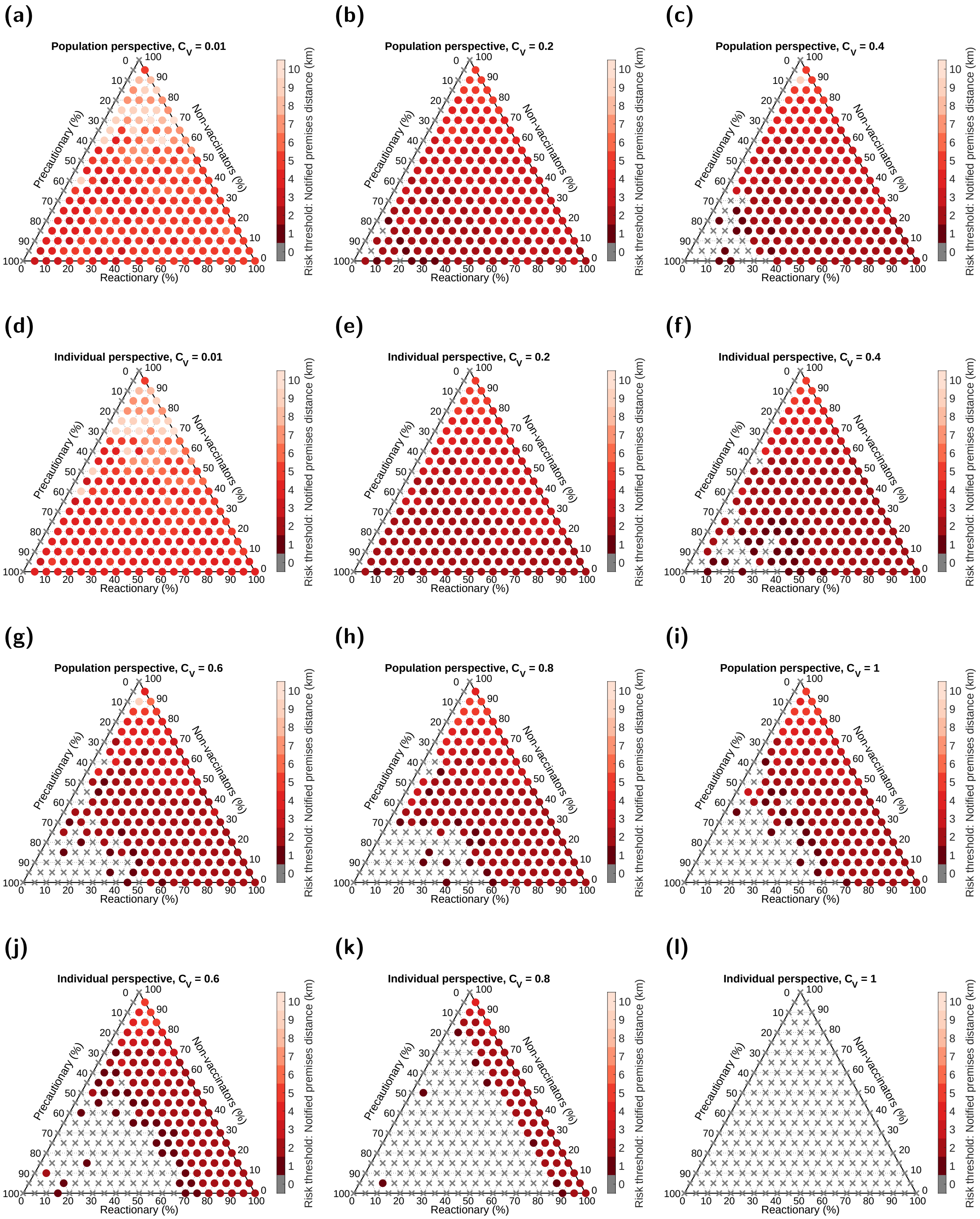

Fig H: Strategy that minimises overall cost in Devon for the 'alternate' pathogen, dependent on the perspective, relative cost of vaccination and vaccine group composition. We performed 500 replicates per scenario. Shading corresponds to the notification distance risk threshold d that returned the lowest median cost across the control scenarios (dark to light corresponding to increases in distance from 1km to 10km). Grey crosses correspond to no reactive vaccination occurring. The ternary plots display optimal strategies from the population and individual perspectives given a relative cost of vaccination, C_V, of: **(a,d)** 0.01; **(b,e)** 0.2; **(c,f)** 0.4; **(g,j)** 0.6; **(h,k)** 0.8; **(i,l)** 1. For an explanation on reading values on a ternary plot, see Fig B.

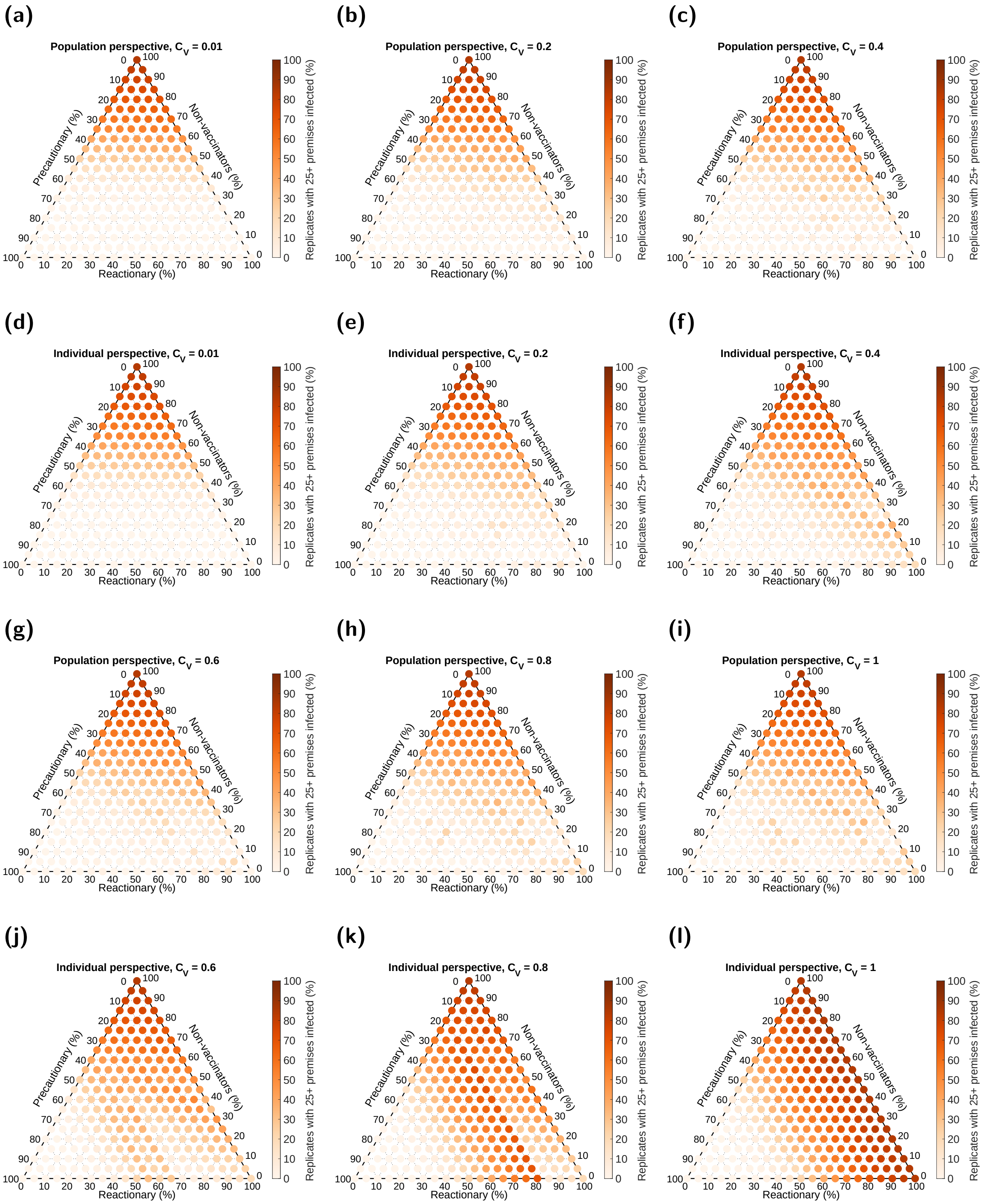

Fig I: Percentage of replicates resulting in 25 or more premises becoming infected under the reactive vaccination strategy minimising the cost in Cumbria ('alternate' pathogen). We performed 500 replicates per scenario. Light to dark shading corresponding to a larger percentage of runs having outbreaks with 25 or more premises infected. The ternary plots display infection outcomes for the reactive vaccination strategy identified as optimal (from population, individual perspectives) given a relative cost of vaccination, C_V, of: **(a,d)** 0.01; **(b,e)** 0.2; **(c,f)** 0.4; **(g,j)** 0.6; **(h,k)** 0.8; **(i,l)** 1. For an explanation on reading values on a ternary plot, see Fig B.

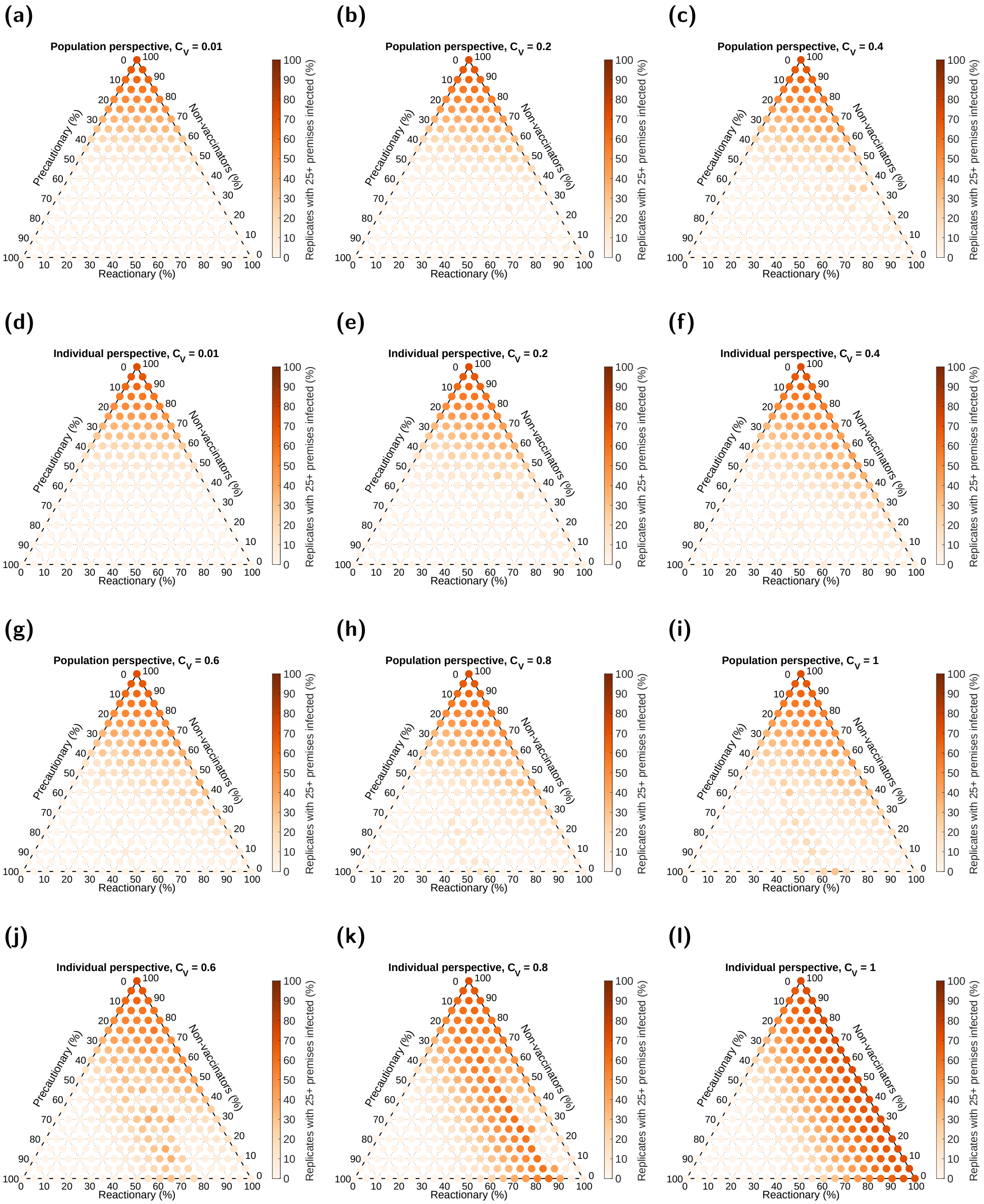

Fig J: Percentage of replicates resulting in 25 or more premises becoming infected under the reactive vaccination strategy minimising the cost in Devon ('alternate' pathogen). We performed 500 replicates per scenario. Light to dark shading corresponding to a larger percentage of runs having outbreaks with 25 or more premises infected. The ternary plots display infection outcomes for the reactive vaccination strategy identified as optimal (from population, individual perspectives) given a relative cost of vaccination, C_V, of: **(a,d)** 0.01; **(b,e)** 0.2; **(c,f)** 0.4; **(g,j)** 0.6; **(h,k)** 0.8; **(i,l)** 1. For an explanation on reading values on a ternary plot, see Fig B.

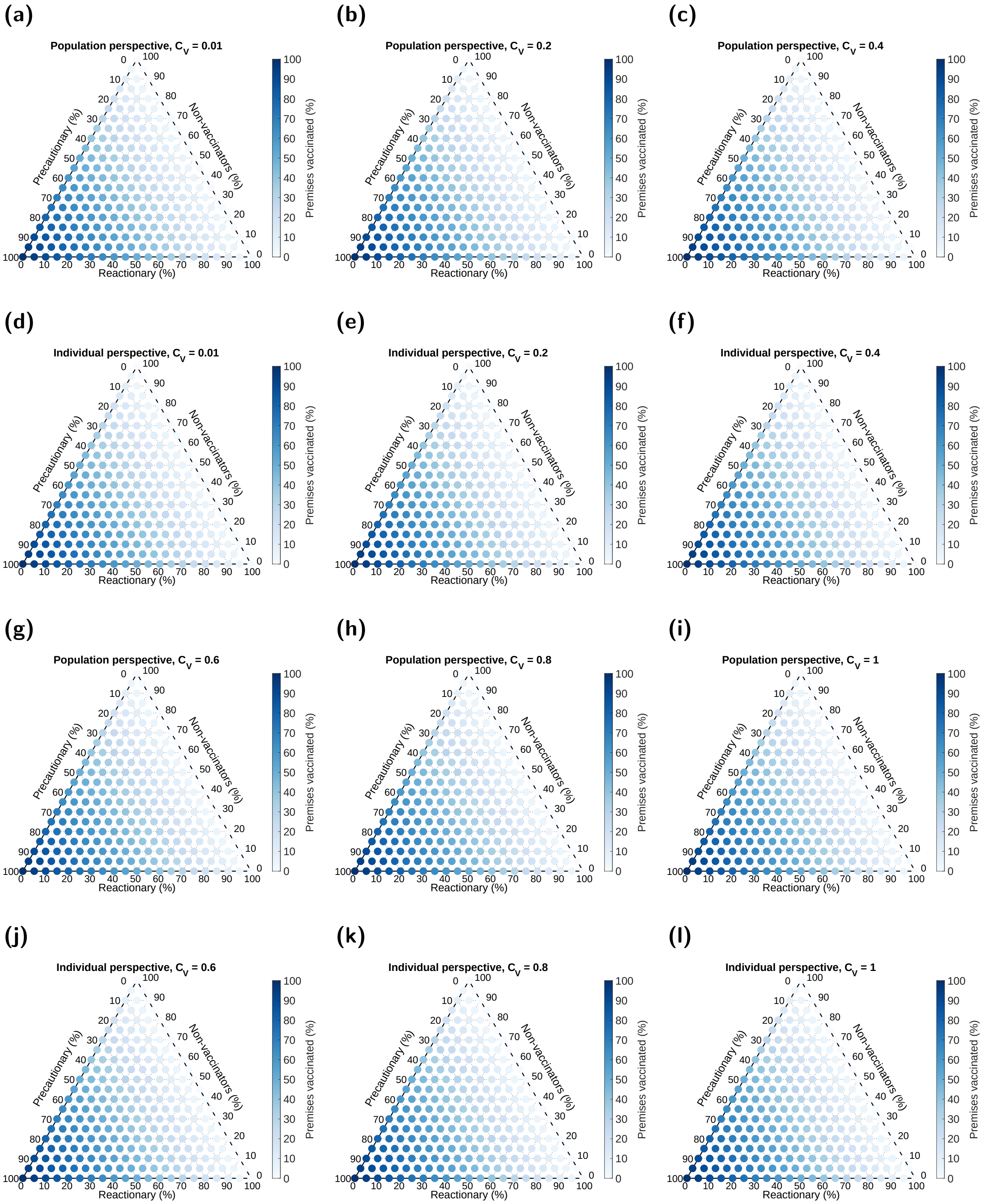

Fig K: Median percentage of premises vaccinated under the reactive vaccination strategy minimising the cost in Cumbria ('alternate' pathogen). We performed 500 replicates per scenario. Light to dark shading corresponding to a larger percentage of premises vaccinated. The ternary plots display vaccination outcomes for the reactive vaccination strategy identified as optimal (from population, individual perspectives) given a relative cost of vaccination, C_V, of: **(a,d)** 0.01; **(b,e)** 0.2; **(c,f)** 0.4; **(g,j)** 0.6; **(h,k)** 0.8; **(i,l)** 1. For an explanation on reading values on a ternary plot, see Fig B.

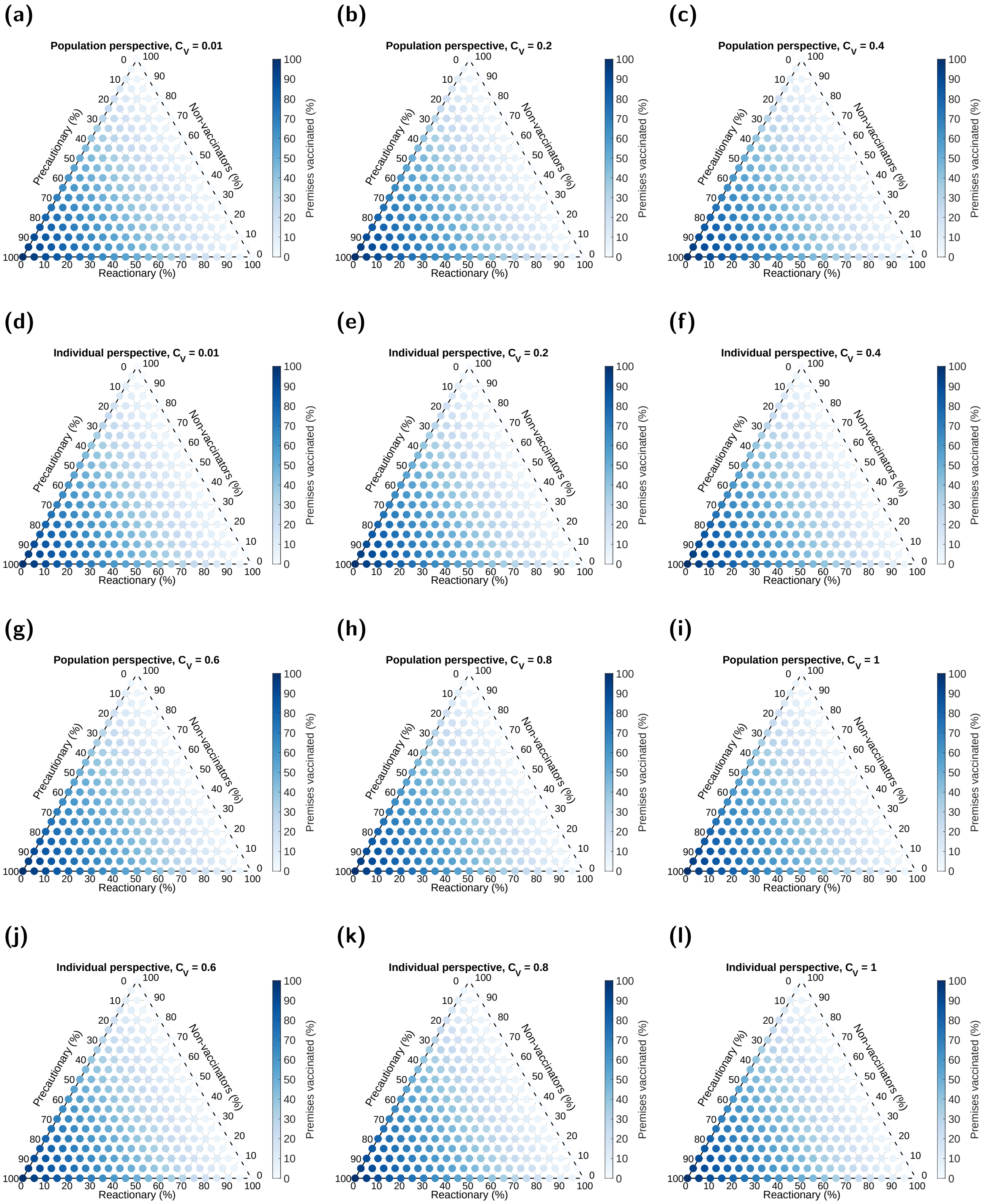

Fig L: Median percentage of premises vaccinated under the reactive vaccination strategy minimising the cost in Devon ('alternate' pathogen). We performed 500 replicates per scenario. Light to dark shading corresponding to a larger percentage of premises vaccinated. The ternary plots display vaccination outcomes for the reactive vaccination strategy identified as optimal (from population, individual perspectives) given a relative cost of vaccination, C_V, of: **(a,d)** 0.01; **(b,e)** 0.2; **(c,f)** 0.4; **(g,j)** 0.6; **(h,k)** 0.8; **(i,l)** 1. For an explanation on reading values on a ternary plot, see Fig B.
