# Supplementary material for: Modelling livestock infectious disease control policy under differing social perspectives on vaccination behaviour
Source: PLoS Comput Biol. 2022 Jul 14;18(7):e1010235. doi: 10.1371/journal.pcbi.1010235 (PMC9282555; doi:10.1371/journal.pcbi.1010235)
Supplement: Attachment — Submitted filename: response_to_reviewers.docx [file pcbi.1010235.s002.docx]

**Response to Reviewers**

**Modelling livestock infectious disease control policy under differing social perspectives on vaccination behaviour** by E. M. Hill, N. S. Prosser, E. Ferguson, J Kaler, M. J. Green, M. J. Keeling & M.J. Tildesley.

*Herein we provide a detailed response to each of the Reviewers comments in blue italics interspersed with the original comments in black font.*

**Where appropriate, at the end of a comment we state in bold red font and within parentheses the line numbers where that edit/addition can be found within the revised manuscript (clean copy and tracked changes copy)**

**Editorial assessment**

Thank you very much for submitting your manuscript "Modelling livestock infectious disease control policy under differing social perspectives on vaccination behaviour" for consideration at PLOS Computational Biology. As with all papers reviewed by the journal, your manuscript was reviewed by members of the editorial board and by several independent reviewers. The reviewers appreciated the attention to an important topic. Based on the reviews, we are likely to accept this manuscript for publication, providing that you modify the manuscript according to the review recommendations.

In particular, I ask that you:

- consider Reviewer #1's comment on the choice of kernel. At a minimum, the point identified by the reviewer should be acknowledged and discussed in the text.

- consider improvements to the abstract and main text conclusions to address Reviewer #2's comment on implications and meaning that can be drawn from this work.

***Authors’ response:***

*Thank you for the positive reception of our manuscript. We are also very appreciative of the steering comments regarding revisions to improve our article. Through our responses to the individual comments raised by the reviewers (detailed below), we believe we have addressed these items in our revised submission.*

**Reviewer 1**

The authors tried to develop a parsimonious modelling framework to combine decision dynamics with epidemiological dynamics. Classically these dynamics are often separately assessed and feed-back ignored. The authors base their efforts on foot-and-mouth outbreaks in two English counties specifying three types of farmers: precautionary, reactionary and non-vaccinators.

The conclusions are substantiated by the results and extensive sensitivity analysis of their model. This of course then relies on the suitability of the model to study the given dynamics.

Intentionally the model is kept fairly simple allowing for robust and tractable results. This illustrates under the model assumptions the potential disagreement between population level and individual level perspective on the most optimal strategy.

The optimal decision to vaccinate for reactionary vaccinators is solely based on the distance to infected farms and costs for vaccination and infection. This requires a full knowledge of the epidemiological state of other farms as well as knowledge on the transmission kernel. Both are often not available during (or directly after) an outbreak. It also does not allow for individual variation in risk attitude. Another draw-back of this approach is that the model does not take into account the influence of peers or influential people on the decision to vaccinate. Non-vaccinators remain non-vaccinators in the model even when all their peers do vaccinate and might potentially pressure non-vaccinators to vaccinate. Also reactionary vaccinators might be persuaded to act differently with many non-vaccinators in their peer-network. For an epizootic infections such as FMD in Europe, learning might not play a role, but the framework does not allow for changes in willingness to vaccinate after having experienced outbreaks. The authors acknowledge this limitation in the discussion.

This modelling framework is, altogether, an important addition in the toolbox of modelling for evaluation of intervention strategies.

***Authors’ response:***

*We thank the reviewer for the detailed appraisal of our study and the kind words on it being a valuable contribution to the literature.*

Some minor remarks remain:

- Why choose for a parameterization of the kernel based on US outbreak? The transmission kernel represents both the infectivity of a particular strain as the contact structure within a country, which is likely to be very different in UK and US.

***Authors’ response:***

*For demonstrating the utility of the model framework, we viewed the specific choice of transmission kernel as not being a primary concern. We found that using the transmission kernel with parameters inferred by Buhnerkempe et al produced a range of epidemic outcomes in both county settings, ideal for the investigative purposes of our study. Additionally, with the UK FMD epidemic occurring in 2001 and our livestock population estimates being from 2020, changes in demography in the past two decades (fewer premises and generally larger herd/flock sizes) can mean using fitted parameters from the 2001 outbreak would not necessarily result in epidemic outbreaks. We have added this disclaimer within the “Model parameterisation“ subsection of the Methods:*

*“We recognise that this parameterisation of the transmission kernel is based on cattle disease outbreaks in the USA, whereas our spatial landscape and livestock epidemiological parameters are informed by data from the UK. We initially investigated using a parameterisation of the transmission kernel inferred from 2001 UK outbreak data. However, with our livestock population estimates being from 2020 and the changes in demography that have occurred during those two decades (fewer premises and generally larger herd/flock sizes), using a parameterisation of the transmission kernel based on 2001 UK outbreak data would not necessarily result in epidemic outbreaks. On the other hand, for demonstrating the utility of the model framework, using the previously described parameterisation for the transmission kernel (with parameters inferred by Buhnerkempe et al.) was ideal for the investigative purposes of our study as it produced a range of epidemic outcomes in both county settings.”*

**(Clean copy: lines 154-164)**

**(Tracked changes: lines 156-167)**

**Reviewer 2**

This is a timely paper, as noted. There has been a call by the infectious disease modelling community, in the human public health field, for greater incorporation of behavioural changes among infectious disease dynamic models.

***Authors’ response:***

*We thank the reviewer for the thoughtful comments provided on our manuscript, which have resulted in us enhancing our discussion of the implications of our findings. We hope the work will be of interest to mathematical modellers and social scientists alike, further promoting interplay between human behaviours and disease epidemic modelling.*

The abstract needs strengthening (beyond ‘deepening our understanding’) in terms of providing recommendations based on the authors findings

***Authors’ response:***

*We have amended the Abstract to improve the messaging to the reader with regards to the implications and meaning that may be drawn from our findings. The ending passage of the Abstract now reads:*

*“Under our assumed epidemiological context, the findings identify livestock disease intervention receptiveness and cost combinations where one would expect strong disagreement between the intervention stringency that is best from the perspective of a stakeholder responsible for supporting the livestock industry compared to a sole livestock owner. Were such discord anticipated and achieving a consensus view across perspectives desired, the findings may also inform those managing veterinary health policy the requisite reduction in intervention cost and/or the required extent of nurturing beneficial community attitudes towards interventions.”*

**(Clean copy: Abstract lines 19-25)**

**(Tracked changes: Abstract lines 19-27)**

The authors note: Population perspective - The population perspective took the viewpoint of an entity who wanted to minimise costs for the overall population. Can this be expanded? For example what is the ‘entity’ and what are the relevant incentives for such an entity?

***Authors’ response:***

*In hindsight, we agree with the reviewer that our original phrasing was ambiguous with regards to what ‘entity’ referred to. We have revised the sentence to now refer to stakeholders and the remit they have, meaning they would be motivated to reduce overall losses and costs across all livestock owners. The revised text now reads:*

*“The population perspective took the viewpoint of a stakeholder responsible for supporting and protecting the livestock industry as a whole, who would therefore seek to minimise costs across all livestock owners.”*

**(Clean copy: lines 214-216)**

**(Tracked changes: lines 217-219)**

There is no mention of the role of incentives given the authors findings. Incentives are likely to be an important discussion point. This should be built into the paper.

From an economic point of view the significance of the differing perspectives (esp the population) in this context needs to be developed (e.g. In other areas of economics, such as health economics, perspective such as the NHS and Societal are relevant.)

***Authors’ response:***

*We thank the author for highlighting these two very relevant points. To incorporate these details we have appended to our paragraph on directions for further study remarks on the significance of differing perspectives, incentivisation of interventions and how we anticipate it may impact our findings:*

*“There are also uncertainties to explore with respect to how the optimum strategy may be affected by the difference between perceived risk (based on farms reporting cases), true risk (based on currently infected farms), and alternative perspectives that include different costs and outcomes in the analysis; parallels may be drawn from public health economics, where the point of view taken for the economic evaluation applied to a specified population can be the health care system (i.e. includes all costs to the health and social care system and, in terms of health outcomes, only considers health-related benefits to patients), whereas the broadest view would be a societal perspective that considers all relevant costs, whoever pays for them, including non-healthcare costs. For example, we are yet to probe how incentivisation of an intervention by a stakeholder responsible for veterinary health would alter our findings. On the basis incentivisation schemes would result in additional non-direct veterinary health related costs for a stakeholder (taking a population-level perspective on control) and a reduced intervention cost to each livestock owner, we anticipate in situations where the relative cost of the intervention is similar to the cost of infection having intervention incentives would result in a convergence in control policy irrespective of perspective taken (due to the optimal spatial range of reactive vaccination likely being: (i) reduced when evaluated from a population-level perspective; (ii) broadened when evaluated from an individual-level perspective).”*

**(Clean copy: lines 523-538)**

**(Tracked changes: lines 528-543)**

Conclusions and recommendations need much more development based on the findings.

***Authors’ response:***

*Supplementing the addition of a paragraph to the Discussion on the implications of differing economic perspectives and implications of incentives, we have also expanded upon what our conclusions mean in terms of recommendations a body responsible for protecting, supporting and improving the livestock industry may want to consider to lessen the prospect of discord between the disease management they advocate and what individual livestock owners would view as optimal for themselves.*

*“These observations raise the question within the veterinary health domain of whether farmers and national-planners should initially adopt a cautious approach in the face of an outbreak with unknown transmission potential, with a recommendation for open dialogue and clear messaging through veterinary health campaigns to culture beneficial community attitudes towards interventions. Equally, our findings indicate (for the assumed epidemiological context) where strong disagreement between the intervention stringency that is best from the perspective of a stakeholder responsible for supporting the livestock industry compared to a sole livestock owner may arise, based on the split of attitudes towards the intervention amongst the population and its relative cost compared to infection. In circumstances where the level of intervention stringency imposed on all the population would be more severe than the control response favourable to a single individual, with pushback expected from the community regarding the intervention stringency, to improve the chances of achieving a consensus across viewpoints one avenue for those managing veterinary health policy would be to lower the cost of the intervention (where possible).”*

**(Clean copy: lines 430-433)**

**(Tracked changes: lines 435-448)**

*The concluding sentence of the article has been revised to the following:
“These insights can aid our understanding of livestock owner reception to official advice during veterinary health emergencies and indicate to bodies that devise veterinary health policy the disease management approach likely to instil unanimity with individual-level viewpoints on what is the appropriate course of disease management.”*

**(Clean copy: lines 559-563)**

**(Tracked changes: lines 565-568)**
